# TDF_CAM_: A method for estimating stable isotope trophic discrimination in wild populations

**DOI:** 10.1002/ece3.9709

**Published:** 2023-01-06

**Authors:** Devin L. Johnson, Michael T. Henderson, Alastair Franke, George J. F. Swan, Robbie A. McDonald, David L. Anderson, Travis L. Booms, Cory T. Williams

**Affiliations:** ^1^ Department of Biology and Wildlife University of Alaska Fairbanks Fairbanks Alaska USA; ^2^ The Peregrine Fund Boise Idaho USA; ^3^ Arctic Raptor Project Rankin Inlet Nunavut Canada; ^4^ Instituto de Conservación, Biodiversidad y Territorio, Facultad de Ciencias Forestales y Recursos Naturales Universidad Austral de Chile Valdivia Chile; ^5^ Environment and Sustainability Institute University of Exeter Cornwall UK; ^6^ Alaska Department of Fish and Game Fairbanks Alaska USA; ^7^ Department of Biology Colorado State University Fort Collins Colorado USA

**Keywords:** animal diet, Bayesian mixing model, nest camera, raptor, stable isotope, trophic discrimination factor, trophic ecology

## Abstract

1. Stable isotope mixing models (SIMMs) are widely used for characterizing wild animal diets. Such models rely upon using accurate trophic discrimination factors (TDFs) to account for the digestion, incorporation, and assimilation of food. Existing methods to calculate TDFs rely on controlled feeding trials that are time‐consuming, often impractical for the study taxon, and may not reflect natural variability of TDFs present in wild populations.
2. We present TDF_CAM_ as an alternative approach to estimating TDFs in wild populations, by using high‐precision diet estimates from a secondary methodological source—in this case nest cameras—in lieu of controlled feeding trials, and provide a framework for how and when it should be applied.
3. In this study, we evaluate the TDF_CAM_ approach in three datasets gathered on wild raptor nestlings (gyrfalcons *Falco rusticolus*; peregrine falcons *Falco perigrinus*; common buzzards *Buteo buteo*) comprising contemporaneous δ^13^C & δ^15^N stable isotope data and high‐quality nest camera dietary data. We formulate Bayesian SIMMs (BSIMMs) incorporating TDFs from TDF_CAM_ and analyze their agreement with nest camera data, comparing model performance with those based on other relevant TDFs. Additionally, we perform sensitivity analyses to characterize TDF_CAM_ variability, and identify ecological and physiological factors contributing to that variability in wild populations.
4. Across species and tissue types, BSIMMs incorporating a TDF_CAM_ outperformed any other TDF tested, producing reliable population‐level estimates of diet composition. We demonstrate that applying this approach even with a relatively low sample size (*n* < 10 individuals) produced more accurate estimates of trophic discrimination than a controlled feeding study conducted on the same species. Between‐individual variability in TDF_CAM_ estimates for ∆^13^C & ∆^15^ N increased with analytical imprecision in the source dietary data (nest cameras) but was also explained by natural variables in the study population (e.g., nestling nutritional/growth status and dietary composition).
5. TDF_CAM_ is an effective method of estimating trophic discrimination in wild animal populations. Here, we use nest cameras as source dietary data, but this approach is applicable to any high‐accuracy method of measuring diet, so long as diet can be monitored over an interval contemporaneous with a tissue's isotopic turnover rate.

## Graphical abstract

Trophic discrimination factors (TDFs) are an important component of stable isotope mixing models (and their use for characterizing wild animal diets) but are difficult to estimate under realistic conditions. We developed a method (TDFCAM) to estimate TDFs in wild populations using stable isotopes in conjunction with a secondary high‐precision diet estimate. We evaluated this approach in three wild raptor datasets, which had contemporaneous stable isotope and nest camera diet data, and found TDFcams to outperform any other TDF tested and produce reliable population‐level estimates of diet composition.

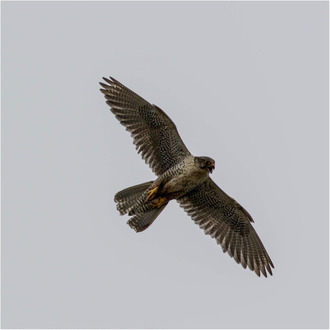

## 1 INTRODUCTION

Stable isotope mixing models (SIMMs) have become a widespread approach for estimating wild animal diet and characterizing trophic dynamics within and among populations. Stable isotope ratios (e.g., of carbon [^13^C/^12^C] and nitrogen [^15^ N/^14^ N], expressed as δ^13^C and δ^15^N values) vary between taxa and are incorporated into consumer tissue from their diet sources in a relatively predictable manner. A consumer's position in isotopic space therefore reflects a combination of the stable isotope values of its diet sources that can be modeled in a Bayesian framework (e.g., *MixSIAR*; Stock et al., 2018) to generate proportional diet estimates that correspond to the temporal window of the isotopic turnover rate of the tissue in question. This Bayesian SIMM (BSIMM) framework allows for the incorporation of prior knowledge of the system (i.e., an “informative prior”) to help guide the model, and a trophic discrimination factor (TDF; expressed as Δ) which accounts for differences in isotope ratios between source and consumer tissue that arise through physiological processes. Although the BSIMM approach has produced reliable diet estimates in empirical comparative studies (e.g., Nifong et al., 2015; Swan et al., 2020), it is highly sensitive to the TDF (Bond & Diamond, 2011). This sensitivity is variable on a case‐by‐case basis and is most pronounced when studies attempt to distinguish point estimates that occupy similar regions in isotopic space (Phillips et al., 2014). An accurate representation of trophic discrimination for the study population is imperative to the successful formulation and interpretation of BSIMMs.

Diet–tissue discrimination is the isotopic shift that occurs when elements from food sources are incorporated into consumer tissue (e.g., McMahon & McCarthy, 2016). Trophic discrimination factors can vary widely by a consumer's diet, growth status, nutritional status, and the tissue being analyzed (reviewed in Martínez del Rio et al., 2009). Although consistent differences in TDFs exist between tissue types (reviewed in Caut et al., 2009), well‐described physiological factors also influence TDFs on an individual basis. Within a population, variability in Δ^15^N is largely associated with an organism's nitrogen use efficiency (related to its nutritional status or stage of growth; e.g., Williams et al., 2007; Hobson et al., 1993), and the protein quality and content of its diet (e.g., Chikaraishi et al., 2015; Kurle et al., 2014). Δ^13^C also varies within a population associated with tissue lipid content and amino acid composition (e.g., Post et al., 2007). It is important to consider these factors in order to estimate TDFs as accurately as possible for a study population.

Existing methods for estimating TDFs have relied on controlled feeding trials, wherein a consumer is maintained on a simple diet with a known isotopic signature, and then the consumer's isotopic values are compared with those of the food it has eaten once isotopic equilibrium between the consumer and its diet has been reached (e.g., Hobson & Clark, 1992; Kurle et al., 2014). In such studies, sample sizes are typically low (5–10 individuals), and individuals are often maintained on a homogeneous diet (Newsome et al., 2010). This controlled approach has been further refined to account for organisms consuming more complex diets by weighting the isotopic values of diet items by their proportional contribution to the overall diet (Greer et al., 2015). However, it is impractical to conduct controlled feeding experiments on all taxa (Martínez del Rio et al., 2009). Instead, studies often rely on previously published TDF values from taxonomically similar species (e.g., Hedd & Montevecchi, 2006; Robinson et al., 2018) or on calculated TDFs based on a compilation of the literature values for their broad taxonomic group of interest (e.g., Caut et al., 2009; Healy et al., 2018). Ultimately, the conditions typical of natural systems are difficult to mimic in a controlled feeding study making the application of laboratory‐derived TDFs to wild populations inappropriate in some circumstances (Swan et al., 2020). This is particularly pertinent when a study population occupies a different life stage or consumes a diet that differs in protein quality or content from the trial population, as these factors have been shown to influence TDFs more than an organism's taxonomy alone (reviewed in: Martínez del Rio et al., 2009). It is thus desirable to estimate TDFs for a wild study population directly, and there is a clear need for a cohesive methodology to do so.

In this study, we validate the TDF_CAM_ approach as an alternative method for estimating trophic discrimination in wild populations (see also: Johnson et al., 2020). Rather than relying on TDF estimates from a controlled feeding study, TDF_CAM_ incorporates proportional diet estimates from a secondary high‐accuracy method for a subset of the study population (in this case, images of prey fed by raptors to their nestlings, collected using a nest camera). These diet estimates (which serve as a proxy for known dietary percentages) can then be used to weight the isotopic values of sources to compare expected vs observed isotopic values of consumers, thereby generating TDFs on an individual basis. This methodology relies on three major assumptions: (1) that the high‐accuracy diet method adequately covers the interval of isotopic turnover for the tissue in question; (2) that the animal's diet remains relatively stable over the course of the monitoring interval; and (3) that each component of the animal's diet is relatively equivalent in protein content and quality. In this study, we formalize and assess the TDF_CAM_ approach and develop a framework for designing studies using TDF_CAM_ for BSIMMs.

Advances in digital imaging technology (e.g., motion‐activated and animal‐borne cameras) have allowed for the accurate estimation of wild animal diet in a broad range of taxa and circumstances over recent years (e.g., the provisioning of altricial seabirds, Edney & Wood, 2020; diet composition of predatory housecats, Loyd et al., 2013). These advances allow for the collection of accurate dietary data of not only provisioning at nests, (Gaglio et al., 2016; Johnson et al., 2020) dens, or caches (Wagnon & Serfass, 2017), but also in free‐ranging taxa as diverse as pinnipeds (Yoshino et al., 2020), sea turtles (Fukuoka et al., 2016), and ungulates (Thompson et al., 2014). Although these methods provide high‐quality fine‐scale dietary data, they are often limited by sample size and their temporal monitoring window (e.g., Johnson et al., 2020). We demonstrate that by incorporating this type of data into the TDF_CAM_ framework, researchers can effectively expand their spatiotemporal scope of inference and ultimately gain a more complete picture of an organism's trophic ecology.

We sought to address three primary research questions: (1) Does the TDF_CAM_ approach improve the accuracy of BSIMM diet estimates? (2) How variable is TDF_CAM_ and what factors contribute to this variability? and (3) What are the key factors to consider when designing a TDF_CAM_ study? We applied the TDF_CAM_ approach to three published datasets that have contemporaneous nest camera and stable isotope data for different raptor species: gyrfalcons (*Falco rusticolus*) in the Alaskan Arctic (Johnson et al., 2020); peregrine falcons (*Falco peregrinus*) in the Canadian Arctic (Robinson et al., 2018); and common buzzards (*Buteo buteo*) in southwest England (Swan et al., 2020). For each species and tissue type, we formulated BSIMMs incorporating calculated TDF_CAM_s and analyzed their agreement with nest camera data, comparing model performance with other relevant TDFs. Additionally, we performed sensitivity analyses to characterize TDF_CAM_ variability, and identified ecological and physiological factors contributing to that variability. Although this study uses nest camera from altricial birds as an example, we outline other types of high‐accuracy diet data that could expand the TDF_CAM_ approach to other methods and taxa. Collectively, our results demonstrate the utility of this approach—introducing a powerful tool that can be used to improve BSIMM performance in wild populations.

## 2 METHODS

### 2.1 Overview of field methods

The three datasets analyzed in this study were quite similar in their methodological approach. Briefly, each study installed motion‐sensitive cameras at raptor nests to monitor nestling diet over their brood‐rearing period. Adults feed prey to nestlings, allowing each diet item to be identified and its biomass estimated to generate proportional estimates of diet over the monitoring period. Nestling tissue was sampled for stable isotope analysis, and each study involved the concurrent sampling of putative prey species for their use as source groups in isotopic mixing models. Generally, these three raptor species exhibit similar life‐history strategies, but with slight differences that are relevant to their growth rate and thus TDF and isotopic turnover rates (Table 1). All three species were sampled for stable isotopes partway through their respective hatch‐fledge intervals: gyrfalcons 26 ± 3 [SD] days of age; peregrine falcons sampled on a weekly basis beginning at 10 days of age; common buzzards 21.5 ± 3 days of age. Tissues were analyzed for δ^13^C and δ^15^N, and diet was estimated for each nestling using BSIMMs and compared with nest camera diet estimates. An overview of sample sizes, tissues sampled, and source groups can be found in Tables S1–S3, and detailed information regarding study systems, ethical approval, and specific methodologies can be found in each of the respective studies (gyrfalcons: Johnson et al., 2020; peregrine falcons: Robinson et al., 2018; common buzzards: Swan et al., 2020). Ethical approval for each of the three raptor datasets was received from their respective appropriate animal ethics committees, and this study did not require additional ethical approval because it did not involve additional fieldwork.

**TABLE 1:** Summary of relevant species‐specific parameters for the three raptor species considered in this study

Species	Clutch size (# eggs laid)	Hatch‐fledge interval	Adult mass (g)	Source
gyrfalcon (*Falco rusticolus*)	3.72 ± 0.71 (Range 1–5)	40–50 days	800–1400 (male) 1000–2100 (female)	Booms et al. (2020)
peregrine falcon (*Falco peregrinus*)	3.0 ± 1.0 (Range 1–5)	30–35 days	524–690 (male) 770–1070 (female)	White et al. (2020)*, Robinson et al. (2018)
common buzzard (*Buteo buteo*)	3.0 ± 1.0 (Range 1–6)	50–60 days	427–1180 (male) 486–1360 (female)	Orta et al. (2020)

* Peregrine falcons vary in body size by region; the presented values are from a population from Nunavut, CA.

### 2.2 Nest camera data

Each of the three studies monitored raptor diet with nest cameras, but differed in their experimental design, camera model/installation, duration and consistency of sampling intervals, and subsequent data analysis and error estimation (Table S4). The gyrfalcon nest camera data continuously covered the interval from when nestlings hatched to when they were sampled for stable isotopes. A small percentage (0.7%) of prey deliveries could not be identified to taxonomic category and were excluded from the analysis. Once each prey delivery was identified and its biomass estimated, a Bayesian bootstrapping protocol was applied to generate error distributions directly comparable to BSIMM output (Johnson et al., 2020).

The peregrine falcon camera dataset covered a comparatively shorter and less continuous interval, ranging from 2 to 16 days per nest (Table S4). Twenty percent of prey deliveries could not be identified to taxonomic category, but each was assigned to its most probable source group and subsequently accounted for in the nest camera diet error structure (following Robinson et al., 2015). Variability in the interval over which nest cameras operated was high; therefore, we identified a subset of three nests with comparatively higher quality camera data than the other nests. These nests with high‐quality data covered 11.0 ± 7.4 days prior to when nestlings were sampled for stable isotopes and had a similar percentage (19%) unknown prey deliveries.

The common buzzard camera dataset similarly had a high degree of variability in coverage intervals (Table S4) and largely represents nestling diet over a time frame after nestlings were sampled for stable isotopes (Swan et al., 2020). 6.1% of prey deliveries were of unknown species, but their biomass could be estimated and proportionally assigned to taxonomic category based on their size and morphological characteristics. 10.6% of prey deliveries by biomass were of species (constituting 21 taxonomic categories) that did not belong to one of the seven isotopic source groups of the study and were excluded from the analysis. Similar to the gyrfalcon study, a Bayesian bootstrapping protocol was applied to generate error distributions (Swan et al., 2020).

### 2.3 TDF_CAM_ methods

We used the following equations to generate a TDF_CAM_ for carbon and nitrogen for each of the three species. Isotope ratios are expressed as δX‰, where δX = [(R_sample_/R_standard_)‐1]*1000, and R is a ratio of heavy: light isotopes of a given element. In the equations, (*i*) represents one of (*n*) prey categories, (*j*) represents an individual in the subset of consumers used (in our case, a nestling), (*m*) is the number of consumers in that subset, and (*P*) is a dietary proportion estimate (in our case, from nest camera analysis):

(1) $${\mathrm{TDF}}_{\mathrm{CAM}}\;\text{Mean}=\frac{\sum_{j=1}^{m}\left[\delta_{j}-\sum_{i=1}^{n}\left(\text{mean}\left(\delta_{i}\right)*P_{\mathrm{ij}}\right)\right]}{m}$$

(2) $$\;{\mathrm{TDF}}_{\mathrm{CAM}}\;\mathrm{SD}=\sqrt{\frac{\sum_{j=1}^{m}{\left(\delta_{j}-{\mathrm{TDF}}_{\mathrm{CAM}}\;\text{Mean}\right)}^{2}\;}{m-1}}\;$$

Because these equations rely on nest camera data, a TDF_CAM_ incorporating the nest camera data from nest X should not be applied to a mixing model for nest X. To avoid doing so, we adjusted raw isotope ratios implementing a jackknifing approach prior to their incorporation into BSIMMs. First, we calculated a mean TDF_CAM_ for each dataset and tissue type, including values from all individuals. For nest X, we calculated a second conditional mean TDF_CAM_ using all individuals in the dataset excluding those from nest X. Then, we adjusted the raw isotope ratios of nestlings at nest X by the difference between the mean TDF_CAM_ and the conditional mean TDF_CAM_. This adjustment process was repeated for all nests, and then BSIMMs were formulated using a mean TDF_CAM_ ± 1 SD calculated for each tissue/species.

We performed sensitivity tests of TDF_CAM_ estimates for each species/tissue to determine the appropriate sample sizes future studies may need to apply this approach. We implemented a Monte Carlo bootstrapping protocol (with 1000 replicates) to estimate the variability around mean TDF_CAM_ estimates when drawing from a randomly selected pool of 1–10 nests.

To explore which factors may influence TDFs in a wild population, we generated a series of linear mixed models for the gyrfalcon dataset with the calculated nitrogen and carbon TDF_CAM_s for each nestling as response variables, *nest* and *year* as random effects, and fixed effects including nestling age, hatch date, sex, body condition (see methods in Table S5), and dietary proportions from nest camera data. We constructed a candidate set of linear mixed models incorporating fixed effects we predicted may influence TDF variability. Models were compared in an AICc framework, and the model set comprising a ∆AICc >2 over the next best model was considered to represent the top model set for each isotope. We only conducted this portion of the analysis on the gyrfalcon dataset because comparable variables (e.g., body condition, hatch date, sex) were unavailable or incomplete for the other two raptor datasets.

### 2.4 Bayesian stable isotope mixing models

We implemented all BSIMMs using the *MixSIAR* package in R (Stock et al., 2018). The input parameters for the models were the raw δ^13^C and δ^15^N values of raptor nestlings that were analyzed against the mean and standard deviations of δ^13^C and δ^15^N values for the set of prey categories relevant to each system. All BSIMMs were formulated with 100,000 iterations thinned by 25 and a burn‐in of 50,000 with three chains and were considered to have converged when they passed the Geweke and Gelman–Rubin Diagnostics (following Stock et al., 2018).

We compared nest camera dietary data against BSIMMs incorporating different TDFs and prior sets using Bhattacharyya's Coefficient (BC). Bhattacharyya's Coefficient is a method of determining overlap between posterior density distributions and has been adopted by stable isotope studies as a statistic to determine method agreement between BSIMMs and other dietary information sources (e.g., Swan et al., 2020) and between different BSIMM outputs (Bond & Diamond, 2011). In the framework of this study, a BC of 1 indicates complete similarity between BSIMM and nest camera diet estimates, and a BC of 0 means, there is no similarity. A BC of >0.60 was considered to represent significant overlap between diet estimates (following Bond & Diamond, 2011). Bhattacharyya's Coefficient values were calculated for each diet item and reported as a mean (±SD) across diet items. For each species and tissue type, a model incorporating a TDF_CAM_ was compared in a BC framework against the top‐performing model from its respective study.

Informative priors are an important factor to consider when testing BSIMM performance and can help guide mixing model output in some instances, particularly when source groups lie between one another in isotopic space (e.g., Phillips et al., 2014; Semmens & Diego, 2018). However, an inappropriate prior may bias model output (e.g., in Johnson et al., 2020; Swan et al., 2020), and BSIMMs formulated with an overly informative prior may simply reflect the source dietary data (e.g., in Robinson et al., 2018). Ideally, an informative prior provides dietary knowledge of the study population under a comparable temporal window (Franco‐Trecu et al., 2013), but does not include high‐precision dietary data directly related to the isotopic study population. Uninformed BSIMMs outperformed informed ones in the gyrfalcon and common buzzard studies, but the peregrine study (Robinson et al., 2018) did not test an informative prior in the same framework—thus, we chose to test peregrine model performance using different TDFs incorporating both informative and uninformative priors. We calculated an informative prior from a previous pellet/prey remain diet study on peregrine falcons in the same study system (Bradley & Oliphant, 1991; Table S2). Prior percentages reflect biomass estimates for individual species binned into four prey categories and were rescaled into Dirichlet hyperparameters before their incorporation into BSIMMs (following Stock et al., 2018).

For the gyrfalcon dataset, we formulated a mixing model with the top‐performing parameters from the Johnson et al. (2020) study: an uninformative prior (where all prey groups are weighted evenly), and a TDF_CAM_ calculated from two nests that were excluded from the rest of the analysis. We formulated a second mixing model using the same input parameters, but using a mean TDF_CAM_ calculated for all 20 nests and adjusted using the jackknifing approach as described above. We formulated mixing models incorporating *Territory* as a random effect and *Year* as a fixed effect to further assess model performance on a finer spatiotemporal scale.

For the peregrine falcon dataset, we compared the performance of two TDF_CAM_s against the TDF applied in the Robinson et al.'s (Robinson et al., 2018) study and assessed the inclusion of an informative prior. We calculated two TDF_CAM_s: one including all individuals in the dataset adjusted using a jackknifing approach and one using individuals from a subset of high‐quality nests (*n* = 4) that were subsequently removed from the analysis (Table S2).

For the common buzzard dataset, we formulated mixing models with the top‐performing parameters from the Swan et al.'s (Swan et al., 2020) study for both feathers and red blood cells (Table S3). We then built mixing models with the same parameters, but applying TDF_CAMs_ calculated for each tissue type using the jackknifing approach incorporating all nests in the dataset (*n* = 20). Although the Swan et al.'s (Swan et al., 2020) study did not test their plasma samples in the same framework, we chose to include plasma in our analysis to test the sensitivity of our approach to different tissue types and temporal windows. We compared plasma diet estimates using a TDF_CAM_ to those from a BSIMM incorporating a TDF from a controlled feeding study on juvenile California condors (Kurle et al., 2013). We did this because the R package Swan et al. (2020) used to generate TDFs for the other tissue types (SIDER; Healy et al., 2018) cannot produce TDF estimates for plasma due to a lack of available data. To assess the cross‐species applicability of the TDF_CAM_ approach, we also formulated BSIMMs using TDF_CAM_s calculated for other species using the same tissue type and tested them in a BC framework (e.g., a peregrine falcon plasma TDF_CAM_ was applied to the common buzzard plasma dataset and vice versa).

## 3 RESULTS

### 3.1 Gyrfalcon dataset

**FIGURE 1:**
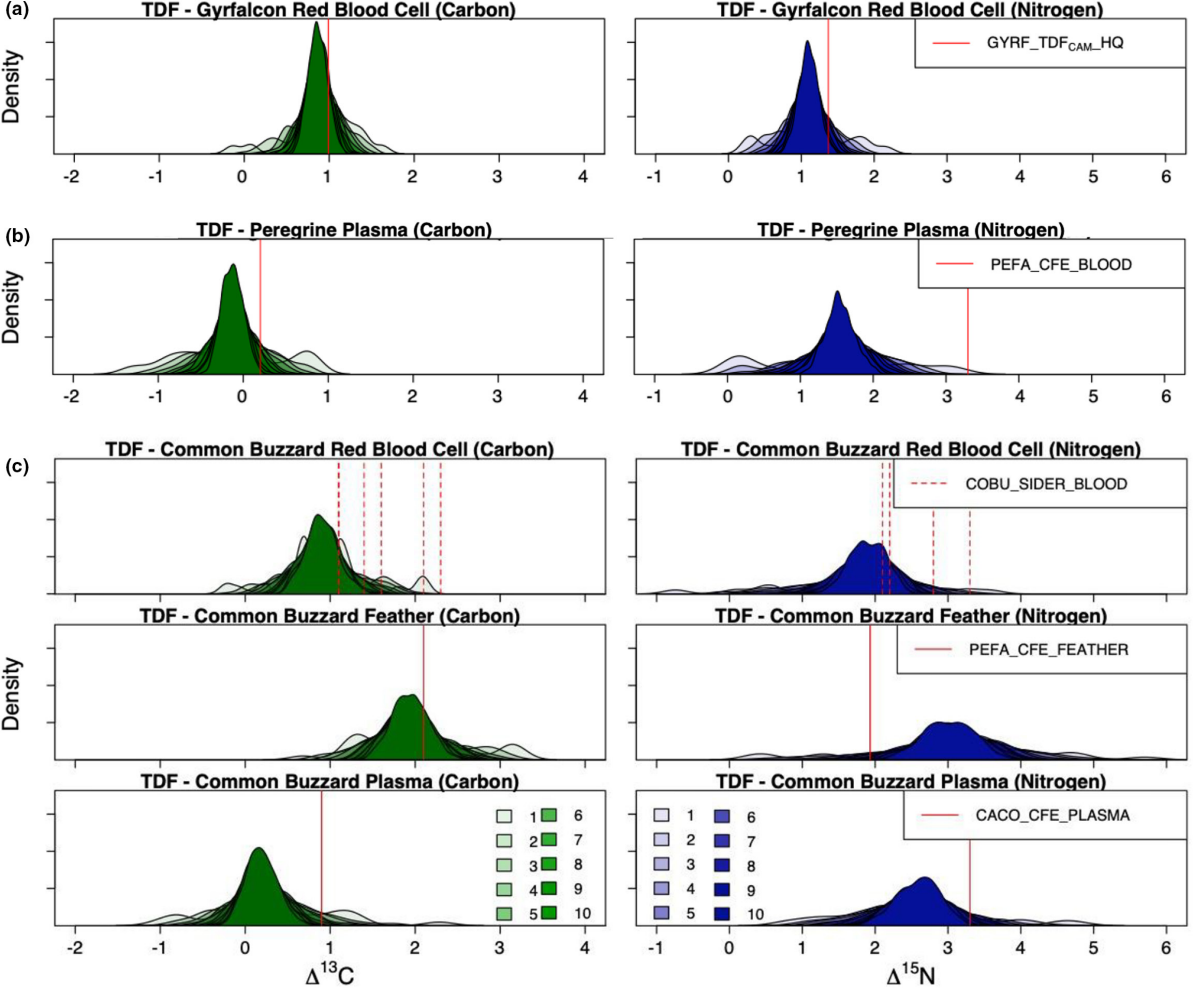
Sensitivity of calculated TDF_CAMs_ to different sample sizes, and comparison against top‐performing TDFs from each respective study. Panel a depicts a TDF_CAM_ derived from gyrfalcon red BLOOD cells; panel B depicts a TDF_CAM_ derived from peregrine falcon PLASMA; and panel C depicts TDF_CAMs_ from common buzzard red BLOOD cells, feathers, and plasma. Polygons (green = carbon, blue = nitrogen) show the density of 1000 bootstrapped estimates of TDF_CAM_ means, randomly sampling nestlings from 1 to 10 nests (shading in legend) from the larger dataset for each raptor. Red lines indicate mean values from the top‐performing TDF in each respective raptor study. In panel a, red lines indicate mean TDF_CAM_ values from the two nests used in the Johnson et al., 2020 study (i.e., GYRF_TDF_CAM__HQ; Table S1). In panel B, red lines indicate mean TDF values from the Hobson and Clark (1992) peregrine falcon feeding experiment (PEFA_CFE_BLOOD; i.e., the TDF used in the Robinson et al., 2018 mixing models; Table S2). In panel C for red blood cells, dashed red lines indicate the mean values of the top‐performing TDF from the Swan et al. (2020) study: Prey‐category‐specific SIDER estimates (COBU_SIDER_BLOOD; Table S3). In panel C for feathers, the red line indicates the mean values of the top‐performing TDF from the Swan et al. (2020) study: A FEATHER TDF from a controlled feeding experiment on peregrine falcons (Hobson & Clark, 1992; PEFA_CFE_FEATHER; Table S3). In panel C for plasma, the red line indicates the mean values of a plasma TDF from a controlled feeding study on California condors (Kurle et al., 2013; CACO_CFE_PLASMA; Table S3).

TDF_CAM_s calculated for each nest of the gyrfalcon dataset ranged from −0.14‰ to 1.63‰ for Δ^13^C and from 0.26‰ to 2.16‰ for Δ^15^N. As we increased the sample size of nests randomly drawn in the Monte Carlo simulation, error estimates around mean TDF_CAM_ values correspondingly decreased for each isotope with an inflection point at five nests (Figure 1a). The jackknifed mean TDF_CAM_ incorporating all gyrfalcon nests had slight differences from the 2‐nest TDF_CAM_ used by Johnson et al., 2020 (Figure 1a), which ultimately improved model performance. The BSIMM formulated with the mean TDF_CAM_ had the best method agreement (BC = 0.76 ± 0.15) and was more consistent with nest camera diet estimates in each year of the study (2017, 2018, 2019; Table S6). However, both TDF_CAM_ approaches (i.e., the high‐quality subset and jackknifing approaches) performed better than any relevant TDF available from the literature or other methods (see Johnson et al., 2020).

**FIGURE 2:**
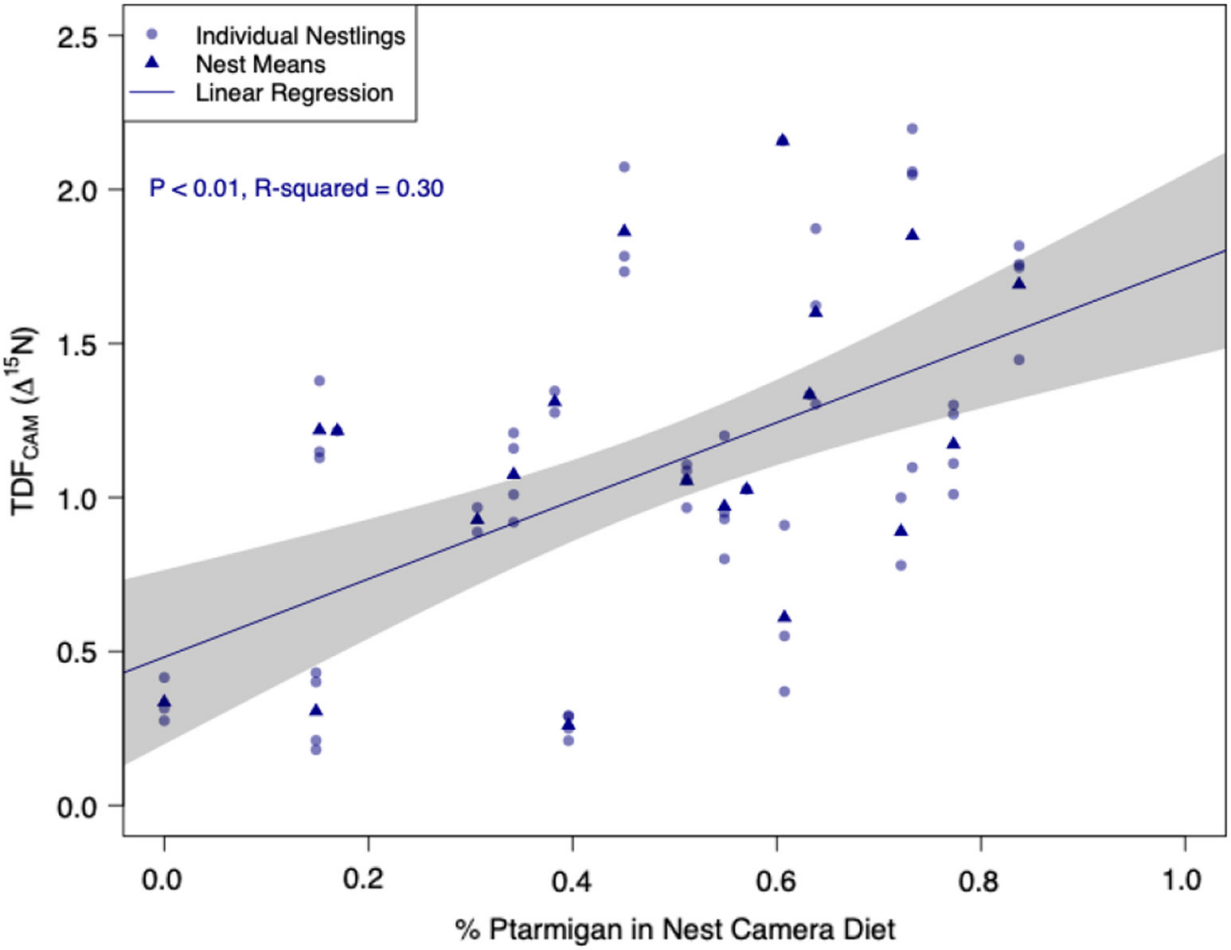
Relationship between calculated nitrogen TDF_CAM_ values (Δ^15^N) and estimated proportion of ptarmigan in the diet (as inferred from nest camera data) for gyrfalcon nestlings on the Seward peninsula (*n* = 57 nestlings in 20 nest‐years, 2016–2019). Shaded polygon represents a 95% confidence interval around the linear regression line.

We observed a positive linear relationship between a nestling's calculated Δ^15^N and the percentage of ptarmigan *Lagopus lagopus* & *L. muta* in their diet as inferred from nest camera data (Figure 2; *p* < .01), which stood out as the single top explanatory variable in an AIC framework (Table S7; ΔAIC = 7.50 over next best model). The top model for Δ^15^N included the following fixed effects: *% Ptarmigan*, (β = 2.04; 95% CI 1.59–2.49), *Nestling Body Condition* (β = −0.01; 95% CI −0.01 to −0.01), *Nestling Age* (β = −0.09; 95% CI −0.14 to −0.04), and *Nestling Hatch Date* (β = 0.04; 95% CI 0.02–0.05; ΔAIC = 7.50 over next best model; Table S7). Although Δ^13^C was not as strongly affected by any individual prey item in the diet, the top model for Δ^13^C included the following fixed effects: *% Arvicoline Rodent* (β = −1.00; 95% CI −2.06 to 0.06), *Nestling Age* (β = −0.06; 95% CI −0.12 to −0.01), and *Nestling Sex* (Female β = 0.16, 95% CI −0.04 to 0.36; Male β = 0.29, 95% CI 0.08–0.50; ΔAIC = 2.76 over next best model; Table S7).

### 3.2 Peregrine falcon dataset

TDF_CAM_s calculated for each nest from the peregrine dataset ranged from −1.29‰ to 0.79‰ for Δ^13^C and from 0.06‰ to 3.12‰ for Δ^15^N. As we increased the sample size of nests randomly drawn from in the Monte Carlo simulation, error estimates around mean TDF_CAM_ values correspondingly decreased for each isotope and continued to decrease at a similar rate up to 10 nests (Figure 1b).

**FIGURE 3:**
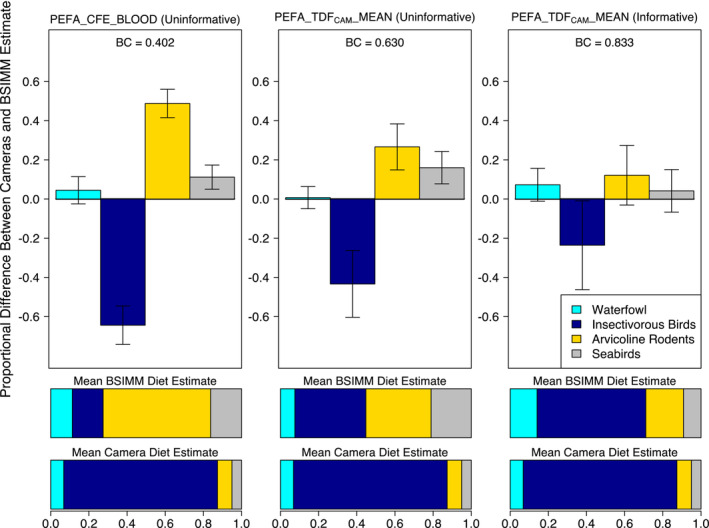
Proportional difference between peregrine falcon Bayesian stable isotope mixing model (BSIMM) diet estimates and nest camera diet estimates by prey category, comparing models constructed with two unique TDFs (details in Table S2). The mixing model output in the left panel incorporates the TDF used in the Robinson et al. (2018) study (PEFA_CFE_BLOOD) and an uninformative prior; the middle panel incorporates the top‐performing TDF_CAM_ from the present study (PEFA_TDF_CAM__MEAN) and an uninformative prior; and the right panel is the top‐performing model incorporating PEFA_TDF_CAM__MEAN and an informative prior. Error bars are ±1 SD. BC is the corresponding MEAN Bhattacharyya's coefficient for each model. Horizontal bars below each plot are the MEAN proportional diet estimates from BSIMM output and nest camera diet estimates for each prey category.

The BSIMM incorporating the mean peregrine TDF_CAM_ and informative priors had the best method agreement with camera data relative to all other TDFs tested (BC = 0.83 ± 0.07; Table S8). Regardless of whether informative priors were incorporated, the TDF_CAM_ calculated for peregrine nesting plasma (PEFA_TDF_CAM__MEAN; Table S2) performed better than a TDF generated from whole blood in a controlled feeding study on adult peregrine falcons (PEFA_CFE_BLOOD; Table S2; Hobson & Clark, 1992). Whereas mixing models applying the peregrine whole blood TDF dramatically overrepresented arvicoline rodents in the diet (Robinson et al., 2018), the TDF_CAM_ approach accurately identified insectivorous birds to be the most important diet item for peregrine falcons (Figure 3). We found that a TDF_CAM_ calculated on plasma from common buzzard nestlings (COBU_TDF_CAM__PLASMA; Table S3) similarly outperformed the controlled feeding study peregrine TDF (Table S8) when applied to the peregrine nestling dataset, highlighting the cross‐species applicability of this approach.

### 3.3 Common buzzard dataset

TDF_CAM_s calculated for each nest of the common buzzard dataset varied by tissue type. Red blood cell TDF_CAM_s among nests ranged from −0.23‰ to 2.11‰ for Δ^13^C and from −0.74‰ to 3.59‰ for Δ^15^N; feather TDF_CAM_s ranged from 0.68‰ to 3.19‰ for Δ^13^C and from 0.42‰ to 5.70‰ for Δ^15^N; and plasma TDF_CAM_s ranged from −1.00‰ to 2.28‰ for Δ^13^C and from 0.79‰ to 4.75‰ for Δ^15^N. Nitrogen TDFs were more variable than those for carbon, and feathers were the most variable tissue type (Figure 1C). Error estimates around mean TDF_CAM_ values continued to decrease as more samples were added to the Monte Carlo simulation, and when 10 nests were incorporated into the equation, the standard deviation was under 0.25‰ for each isotope and tissue type (Figure 1C).

**FIGURE 4:**
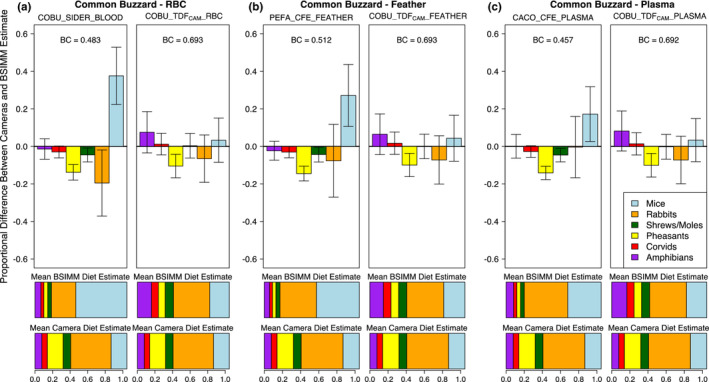
Proportional difference between common buzzard Bayesian stable isotope mixing model (BSIMM) diet estimates and nest camera diet estimates by prey category, comparing models constructed with two unique TDFs for each tissue type: Red blood cells (a); feather (b) and plasma samples (c, details in Table S3). For each tissue type, the mixing model output in the left panel incorporates the top‐performing TDF from the Swan et al. (2020) study, and the right panel incorporates the top‐performing TDF_CAM_ from the present study. Plasma samples were not tested in the same framework in the Swan et al., 2020 study, so we included the next best available TDF from the literature for comparison (Table S3). Error bars are ±1 SD. BC is the corresponding Bhattacharyya's coefficient for each model from Table S9. Horizontal bars below each plot are the mean proportional diet estimates from BSIMM output and nest camera diet estimates for each prey category.

Common buzzard BSIMMs incorporating TDF_CAM_s had better method agreement with nest camera data than any other available TDF for each tissue type (Table S9). For red blood cells, the TDF_CAM_ model outperformed the top model from the Swan et al.'s (Swan et al., 2020) study (COBU_SIDER_BLOOD; Table S3.3), and more accurately represented the proportions of the two most commonly used diet items (rabbits & mice; Figure 4a). Our diet estimates using a SIDER TDF differ slightly from those in the Swan et al.'s (Swan et al., 2020) study due to methodological differences in BSIMM implementation (i.e., including *Territory and Nest* effects in *MixSIAR*), but we also found a TDF_CAM_ to outperform their top TDF when using the same exact methodological approach (Figure S1). We found that a TDF_CAM_ calculated from gyrfalcon nestling red blood cells (GYRF_TDF_CAM__MEAN; Table S1) similarly outperformed the top red blood cell model from the Swan et al.'s (Swan et al., 2020) study (Table S9). For feathers, the TDF_CAM_ model had marginally better fit with camera data than the top model from the Swan et al.'s (Swan et al., 2020) study (PEFA_CFE_FEATHER; Table S3), and generally produced similar diet estimates (Figure 4b). For plasma, the TDF_CAM_ model had better method agreement than a model incorporating a TDF from a controlled feeding study on juvenile California condors (CACO_CFE_PLASMA; Table S3; Figure 4c).

## 4 DISCUSSION

For all species and tissue types considered in this study, the TDF_CAM_ approach resulted in better method agreement with high‐accuracy dietary (nest camera) data than any other available TDF. Although we observed a high degree of variability in TDFs calculated at the individual level, this variation was not random. We found that TDF estimates were particularly sensitive to an individual's diet and growth/nutritional status, as others have reported (Caut et al., 2009; Kurle et al., 2014; Williams et al., 2007). In exploring these three datasets, we identify three factors that influence the accuracy and applicability of the TDF_CAM_ approach: the accuracy of the dietary source data; whether the time frame of dietary source data matches the interval of isotopic incorporation for a given tissue; and the adequate inclusion and characterization of isotopic source groups. With these factors in mind, we provide practical advice for designing studies using TDF_CAM_s and BSIMMs.

Dietary estimates from stable isotope mixing models are highly sensitive to the TDF that is applied to them (e.g., Swan et al., 2020). Variation in mean TDF estimates on the scale of <1‰ in some circumstances can have significant effects on reconstructed diet estimates and their conservation and management implications (Bond & Diamond, 2011). We observed large differences between TDF_CAM_s and TDFs generated from controlled feeding studies, and ultimately the TDF_CAM_ approach improved the accuracy of modeled diet estimates in each instance.

The performance and accuracy of a TDF_CAM_ is inherently dependent on the quality of the dietary source data it incorporates. Raptor nest camera dietary data are highly accurate so long as cameras are installed correctly and most diet items fed to nestlings can be identified (and their biomass estimated; e.g., Robinson et al., 2019). The three nest camera datasets varied in their camera settings and study design, which ultimately influenced their accuracy (% unidentified prey) and their monitoring intervals (Table S4). When calculating a TDF, whether in a controlled feeding study or in a wild population, it is important to ensure that an individual's diet is closely monitored or held constant for an interval contemporaneous with the isotopic turnover rate of the tissue in question (e.g., Hobson & Clark, 1992). This is particularly pertinent in wild animal populations, where diet shifts often occur. For example, gyrfalcon diet on the Seward Peninsula changes across the brood‐rearing period from predominantly ptarmigan to predominantly Arctic ground squirrels (Robinson et al., 2019). If diet is only monitored for a fraction of the tissue's isotopic incorporation interval, it may result in larger differences between observed and predicted isotope ratios driving incorrect estimates of trophic discrimination, particularly if diet was variable over that interval. Of the three datasets for which we applied the TDF_CAM_ approach, the gyrfalcon camera interval (~25 days from hatch to sampling date) best matched the turnover rate for the tissue in question (red blood cells, half‐life ~2 weeks; Phillips & Eldridge, 2006) and accordingly had lower variability in Δ^13^C & Δ^15^N (Figure 1a). We suspect that variability in the monitoring intervals of the other two datasets (Table S4) contributed to both their relatively larger variance in TDF estimates (Figure 3) and differences between BSIMM and nest camera diet estimates (Figure 4). We suggest that future studies match monitoring intervals to isotopic turnover rates as closely as possible.

Isotopic turnover occurs in different tissues at different rates, allowing stable isotope studies to utilize multiple tissue types from an individual to represent its diet over different time frames (e.g., Phillips & Eldridge, 2006). Since different tissues also have consistently different TDFs (reviewed in Caut et al., 2009), studies must either account for multiple tissues in their controlled feeding experiment design (e.g., Hobson & Clark, 1992) or apply TDFs from multiple sources (e.g., Swan et al., 2020). One major benefit of the TDF_CAM_ approach is that it can be applied to multiple tissue types simultaneously. In the common buzzard dataset, we show that a TDF_CAM_ outperforms other TDFs for each tissue type: plasma, red blood cells, and feathers (Table S4 and Figure 4). Differences in estimated TDFs followed the expected relationships (from Caut et al., 2009), with feathers exhibiting higher Δ^13^C and Δ^15^N than the other two tissue types. These three tissue types represent different time periods of isotopic incorporation, with estimated half‐lives ranging from several days (plasma) to multiple weeks (feathers; Phillips & Eldridge, 2006), and BSIMMs using TDF_CAM_s produced similar diet estimates across tissue types (Figure 4). Differences in bulk Δ^13^C and Δ^15^N between tissues of the same organism are often driven by differences in tissue‐specific amino acid composition (e.g., McMahon & McCarthy, 2016); thus, we recommend calculating a TDF_CAM_ directly for the tissue type in question, and closely considering that tissue's isotopic turnover rate. With continuous high‐quality dietary source data and accurate estimates of isotopic turnover, future studies may further refine this approach by editing the monitoring interval to match tissue‐specific turnover rates. Specifically, we propose adjusting the proportional weight of the dietary source data throughout the monitoring period using an exponential decay function incorporating estimated turnover rates for the tissue types in question to potentially improve the accuracy of seasonal dietary comparisons within a study population.

Although diet estimation was best when using a species‐specific TDF_CAM_, we found that a TDF_CAM_ developed for the same tissue on nestlings of a different species (at a similar stage of development) can outperform a TDF calculated from a controlled feeding study on adults of the same species as the focal organism. This is exemplified in the peregrine falcon dataset, where the mean Δ^15^N was 3.17‰ lower in our estimates than the value from a controlled feeding trial (Figure 1b), resulting in very different diet estimates (Figure 3). We offer two complementary physiological explanations for this large discrepancy in the peregrine nestling Δ^15^N TDF_CAM_ (plasma) and TDF estimates from a controlled feeding study on adult peregrine falcons (whole blood; Hobson & Clark, 1992). First, growing individuals have higher nitrogen use efficiency relative to nongrowing individuals, resulting in lower Δ^15^N (Hobson et al., 1993)—thus, the application of a TDF developed in controlled feeding trials using adults to studies involving rapidly growing nestlings is inappropriate. Second, the initial peregrine study used plasma, but applied literature TDF values derived from whole blood, which is problematic because plasma and whole blood have consistently different TDFs across taxa (e.g., Caut et al., 2009; Hahn et al., 2012). Thus, we suggest that considering factors that influence trophic discrimination is particularly important when selecting an appropriate TDF. Ideally, one would use a TDF that is formulated for both the consumer species and appropriate life‐history stage for the study population in question, and the TDF_CAM_ method facilitates this approach when a species‐ and age class‐specific controlled feeding experiment is unavailable or impractical.

Another important factor influencing the performance of the TDF_CAM_ method (and BSIMMs in general) is the adequate inclusion and isotopic characterization of source groups (Phillips et al., 2014). Since TDF_CAM_s are calculated based on the isotopic values of source groups (Equations 1 and 2), it is integral that each main source group is accounted for in the mixing space. Because there is geographic and seasonal variation in isotopic values across taxa (e.g., Hobbie et al., 2017), we recommend source groups be sampled from the same study site as consumers at a comparable time interval, and multiple samples be attained from each species to account for variability in the diet, sex, and age classes of prey groups. It becomes more difficult to account for each source group as the prey base widens, as exemplified in the common buzzard dataset. Common buzzards are dietary generalists (e.g., Swan et al., 2020) and had a wider prey base than the two relatively specialized Arctic species. 10.6% of the Common buzzard diet was comprised of species that were not assigned to an isotopic source group (e.g., passerines, squirrels, and pigeons; Swan et al., 2020). Accordingly, Common buzzard BSIMMs had lower method agreement with camera data overall than the other two datasets—perhaps driven by prey groups that were not represented in the mixing space and contributing to TDF_CAM_ variability. Thus, although some of the observed variability in TDF_CAM_ estimates may be rooted in analytical imprecision in nest camera estimates, monitoring intervals mismatched with isotopic incorporation windows, or inadequate sampling of source groups, some may also arise from natural variability in ecological and physiological factors influencing the study populations.

Wild populations express a wide degree of interindividual variability in dietary composition and quality (Bolnick et al., 2003) along with other physiological factors (e.g., growth rate and nutritional stress) that may contribute to natural variability in TDF values (e.g., Newsome et al., 2010). In the gyrfalcon dataset, we found that variability in Δ^15^N was best explained by the percentage of ptarmigan in the diet, along with a nestling's body condition, age, and hatch date (Table S5). One possible explanation for the positive relationship between the ptarmigan content of a gyrfalcon nestling's diet and their nitrogen TDF (Figure 2) may relate to protein catabolism, as ptarmigan have very low body fat (constituting 2%–3% of their total body mass; Thomas, 1982), and thus higher protein content relative to other species comprising the raptor prey base. Trophic discrimination is altered by protein catabolism, such that individuals with protein‐rich diets (i.e., leaner diets) are predicted to have reduced nitrogen‐use efficiency and increased Δ^15^N as they catabolize protein for energy (Martínez del Rio et al., 2009). Alternatively, this relationship may stem from analytical imprecision in biomass estimates for ptarmigan in the nest camera data or biased isotopic characterization of the ptarmigan endpoint, although the Johnson et al.'s (Johnson et al., 2020) study attempted to represent these values as accurately as possible. We also found higher Δ^15^N in individuals that were growing more slowly (i.e., with lower body condition) and slightly higher Δ^15^N in younger nestlings, both of which are predicted based on effects on an organism's nitrogen use efficiency (Martínez del Rio et al., 2009). Across many avian taxa, individuals that hatch earlier routinely have higher fitness and survival (e.g., Harriman et al., 2017); thus, we also included hatch date in our models, which may be correlated with nestling body condition and growth rate. We found that variability in Δ^13^C was best explained by the percentage of arvicoline rodents in the diet, along with a nestling's age and sex. Organisms consuming more lipid‐rich diets have been found to exhibit lower Δ^13^C in wild populations (e.g., Newsome et al., 2010). Gyrfalcon nestlings selectively consume muscle tissue from larger prey items (e.g., ptarmigan and Arctic ground squirrel), but consume arvicoline rodents whole (Robinson et al., 2019), potentially consuming a higher proportion of lipid‐rich organs when arvicoline rodents feature predominantly in their diet. Nestlings of different ages and sexes may also have consistent differences in the lipid content of their tissue (e.g., Blem, 1976), reflecting the patterns we observed in our analysis. Because wild populations are so variable on an interindividual basis in traits that influence TDFs, the TDF_CAM_ approach may be more effective at encompassing that variability than controlled feeding experiments.

The TDF_CAM_ approach requires a secondary source of high‐quality dietary data for a subset of the study population, which can then be applied to a larger isotopic dataset (e.g., Johnson et al., 2020). Because high‐quality dietary data are typically costly and logistically difficult to collect, sample size is a key consideration for this approach. We present two options researchers may consider when designing a TDF_CAM_ study depending on the quality and consistency of dietary data available. A researcher may elect either to calculate a TDF_CAM_ using the highest quality subset of their dietary data or apply a jackknifing approach to incorporate the entire dataset. Johnson et al. (2020) demonstrated that carefully choosing a small subset of the highest quality gyrfalcon camera data (*n* = 2 nests) that also represented natural variability in the system (i.e., different diets and sampling ages) resulted in TDFs which outperformed any other available. Calculating a mean TDF_CAM_ incorporating all gyrfalcon nests (*n* = 20) and applying a jackknifing approach did marginally improve model fit across all years of the study (Table S3). Similarly, the mean model (*n* = 14 nests) outperformed the high‐quality model (*n* = 4 nests) for the peregrine falcon dataset (whether or not an informative prior was applied), but both TDF_CAM_ methods produced more realistic diet estimates than the TDF from a controlled feeding experiment on adult peregrine falcons. Reliable TDF_CAM_s can thus be generated with reasonably small sample sizes (i.e., 2–4 nests), but adding more samples will ultimately better characterize the natural isotopic variability of the study population.

Although we developed the TDF_CAM_ approach using raptor nest cameras for high‐quality dietary source data, it is broadly applicable to other taxa and methods of diet study, but not necessarily to all study species and situations. This study is not the first to estimate TDFs in wild populations using a secondary method of dietary data, although it is uncommon. For instance, Newsome et al. (2010) applied a similar method to estimate Δ^13^C and Δ^15^N in a wild population of California sea otters (*Enhydra lutris nereis*) using high‐quality observational data. Bradley et al. (2015) similarly used stomach contents in conjunction with stable isotopes to roughly calculate the Δ^15^N of individual amino acids and ultimately estimate trophic position in wild marine teleost populations. Clearly, there are advantages to estimating TDFs in wild populations across a broad range of taxa, and the TDF_CAM_ approach is a robust method to do so, provided high‐quality dietary estimates are attainable for a subset of the study population. In this study, we apply it to nest camera data for three raptor species, and thus it appears the TDF_CAM_ approach may work best for altricial bird species. Theoretically, however, this approach could be used with any method of diet study that can accurately cover an interval contemporaneous with an isotopic incorporation rate. Observational methods (e.g., nest cameras, direct observations, and camera collars) provide high‐quality dietary data over longer and more controllable intervals than indirect methods of diet study (e.g., pellet/prey analysis and stomach/fecal contents), and are thus better suited to the TDF_CAM_ approach. However, indirect methods that allow for accurate repeat sampling (e.g., DNA metabarcoding and next‐generation sequencing) also show promise. As modern advances in technology and techniques improve the accuracy of fine‐scale diet estimates, the TDF_CAM_ approach provides a powerful tool to improve the performance of BSIMMs in wild populations.

## AUTHOR CONTRIBUTIONS

**Devin L. Johnson:** Conceptualization (lead); data curation (lead); formal analysis (lead); funding acquisition (equal); investigation (lead); methodology (lead); project administration (equal); visualization (lead); writing – original draft (lead); writing – review and editing (equal). **Michael T. Henderson:** Data curation (supporting); funding acquisition (supporting); writing – review and editing (equal). **Alastair Franke:** Data curation (supporting); writing – review and editing (equal). **George Swan:** Data curation (supporting); writing – review and editing (equal). **Robbie A. McDonald:** Data curation (supporting); writing – review and editing (equal). **David L. Anderson:** Funding acquisition (supporting); writing – review and editing (equal). **Travis Booms:** Data curation (supporting); funding acquisition (supporting); writing – review and editing (equal). **Cory Williams:** Conceptualization (supporting); funding acquisition (supporting); methodology (supporting); supervision (equal); writing – review and editing (equal).

## CONFLICT OF INTEREST

The authors declare that there is no conflict of interest.

## Supporting information


Data S1.

## Data Availability

The three underlying datasets we analyzed can be found attached to their respective studies (gyrfalcons: Johnson et al., 2020; peregrine falcons: Robinson et al., 2018; common buzzards: Swan et al., 2020). Additionally, the code we used to analyze them will be made available on the Dryad digital repository.
