# Supplementary material for: TDFCAM: A method for estimating stable isotope trophic discrimination in wild populations
Source: Ecol Evol. 2023 Jan 6;13(1):e9709. doi: 10.1002/ece3.9709 (PMC9817186; doi:10.1002/ece3.9709)
Supplement: Supplementary file 1 — Data S1. [file ECE3-13-e9709-s001.docx]

TDF_CAM_: A NOVEL METHOD FOR ESTIMATING TROPHIC DISCRIMINATION IN WILD POPULATIONS

**Supplemental Material**

Table S1: Metadata for the gyrfalcon dataset and input parameters for BSIMMs.

| **GYRFALCON DATASET** | | | | | | | | | | | | | | |
| --- | --- | --- | --- | --- | --- | --- | --- | --- | --- | --- | --- | --- | --- | --- |
| **Species** | **Location** | | **Years** | | **Tissue Type** | **# Nests (# Nestlings)** | | **# Source Groups** | | **# Prey Tissue Samples (# species)** | | | **Literature Source** | |
| Gyrfalcon | Seward Peninsula, Alaska | | 2016–2019 | | RBC | 20 (57) | | 5 | | 185 (10) | | | Johnson et al. 2020 | |
| **SOURCE GROUPS** | | | | | | | | | | | | | | |
| **Category** | | | | | **δ^13^C ﻿(‰) ± SD** | | | | **δ^15^N﻿ (‰) ± SD** | | | **# Samples** | | |
| Arctic Ground Squirrel | | | | | -25.30 ± 0.49 | | | | 3.07 ± 1.04 | | | 43 | | |
| Insectivorous Bird | | | | | -24.34 ± 1.22 | | | | 5.49 ± 1.72 | | | 28 | | |
| Arvicoline Rodent | | | | | -26.91 ± 0.69 | | | | 3.25 ± 0.84 | | | 29 | | |
| Jaeger | | | | | -23.35 ± 1.78 | | | | 8.36 ± 2.07 | | | 20 | | |
| Ptarmigan | | | | | -24.40 ± 0.54 | | | | 1.83 ± 0.77 | | | 64 | | |
| **TROPHIC DISCRIMINATION FACTORS** | | | | | | | | | | | | | | |
| **TDF Name** | | **Tissue Type** | | **Category** | | | **∆^13^C (± SD)** | | | | **∆^15^N (± SD)** | | | **Source** |
| GYRF_TDF_CAM_ _HQ | | RBC | | TDF_CAM_  (2 high-quality nests) | | | 0.99 ± 0.17 | | | | 1.37 ± 0.22 | | | Johnson et al. 2020  (Top Model) |
| GYRF_TDF_CAM__MEAN | | RBC | | TDF_CAM_  (20-nest mean) | | | 0.87 ± 0.46 | | | | 1.09 ± 0.55 | | | Present Study |

Table S2: Metadata for the peregrine falcon dataset and input parameters for BSIMMs.

| **PEREGRINE FALCON DATASET** | | | | | | | | | | | | | | | | | | |
| --- | --- | --- | --- | --- | --- | --- | --- | --- | --- | --- | --- | --- | --- | --- | --- | --- | --- | --- |
| **Species** | **Location** | | **Years** | | | **Tissue Type** | | **# Nests (# Nestlings)** | | | **# Source Groups** | | | **# Prey Tissue Samples (# species)** | | | | **Literature Source** |
| Peregrine Falcon | Rankin Inlet, Nunavut | | 2010–2012 | | | Plasma | | 14 (37) | | | 4 | | | 79 (15) | | | | Robinson et al. 2018 |
| **SOURCE GROUPS** | | | | | | | | | | | | | | | | | | |
| **Category** | | | | **δ^13^C ﻿(‰) ± SD** | | | | | | **δ^15^N﻿(‰) ± SD** | | | | | **# Samples** | | | |
| Duck | | | | -19.56 ± 0.62 | | | | | | 9.31 ± 2.67 | | | | | 6 | | | |
| Insectivorous Bird | | | | -23.30 ± 1.31 | | | | | | 6.60 ± 1.75 | | | | | 35 | | | |
| Arvicoline Rodent | | | | -25.06 ± 0.46 | | | | | | 0.96 ± 0.77 | | | | | 14 | | | |
| Seabird | | | | -18.99 ± 0.77 | | | | | | 15.97 ± 1.15 | | | | | 20 | | | |
| **TROPHIC DISCRIMINATION FACTORS** | | | | | | | | | | | | | | | | | | |
| **TDF Name** | | **Tissue Type** | | | **Category** | | | | **∆^13^C (± SD)** | | | | **∆^15^N (± SD)** | | | | **Source** | |
| PEFA_CFE_BLOOD | | Whole Blood | | | Controlled Feeding Experiment (Adult Peregrines) | | | | 0.20 ± 0.01 | | | | 3.30 ± 0.40 | | | | Hobson & Clark 1992 (used in Robinson et al. 2018) | |
| PEFA_TDF_CAM__HQ | | Plasma | | | TDF_CAM_ (4 high-quality nests) | | | | 0.26 ± 0.58 | | | | 1.91 ± 0.98 | | | | Present Study | |
| PEFA_TDF_CAM__MEAN | | Plasma | | | TDF_CAM_ (14-nest mean) | | | | -0.12 ± 0.71 | | | | 1.55 ± 0.98 | | | | Present Study | |
| **INFORMATIVE PRIORS** | | | | | | | | | | | | | | | | | | |
| **Duck** | | **Insectivorous Bird** | | | | | **Arvicoline Rodent** | | | | | **Seabird** | | | | **Source** | | |
| 0.35 | | 0.35 | | | | | 0.26 | | | | | 0.04 | | | | Bradley and Oliphant, 1991 | | |

Table S3: Metadata for the common buzzard dataset and input parameters for BSIMMs.

| **COMMON BUZZARD DATASET** | | | | | | | | | | | | | | | |
| --- | --- | --- | --- | --- | --- | --- | --- | --- | --- | --- | --- | --- | --- | --- | --- |
| **Species** | **Location** | **Years** | | **Tissue Type** | | | **# Nests (# Nestlings)** | **# Source Groups** | | | **# Prey Tissue Samples (# species)** | | | **Literature Source** | |
| Common Buzzard | Southern England | 2015 | | RBC; Feathers; Plasma | | | 20 (29) | 6 | | | 69 (12) | | | Swan et al. 2020 | |
| **SOURCE GROUPS** | | | | | | | | | | | | | | | |
| **Category** | | | | | | **δ^13^C ﻿(‰) ± SD** | | | | **δ^15^N﻿(‰) ± SD** | | | **# Samples** | | |
| Amphibian | | | | | | -26.54 ± 0.44 | | | | 6.25 ± 1.47 | | | 7 | | |
| Corvid | | | | | | -24.97 ± 0.58 | | | | 8.60 ± 1.53 | | | 5 | | |
| Gamebird | | | | | | -24.71 ± 2.13 | | | | 6.34 ± 0.73 | | | 9 | | |
| Insectivorous Mammal | | | | | | -25.77 ± 1.01 | | | | 9.02 ± 1.73 | | | 7 | | |
| Rabbit | | | | | | -28.76 ± 0.52 | | | | 6.11 ± 1.69 | | | 24 | | |
| Small Mammal | | | | | | -28.33 ± 1.49 | | | | 4.19 ± 2.59 | | | 17 | | |
| **TROPHIC DISCRIMINATION FACTORS** | | | | | | | | | | | | | | | |
| **TDF Name** | | | **Tissue Type** | | **Category** | | | | **∆^13^C (± SD)** | | | **∆^15^N (± SD)** | | | **Source** |
| COBU_SIDER_BLOOD | | | Whole Blood | | SIDER (Amphibian)  SIDER (Corvid)  SIDER (Gamebird)  SIDER (I. Mammal)  SIDER (Rabbit)  SIDER (S. Mammal) | | | | 1.60 ± 1.60  1.10 ± 1.50  1.10 ± 1.50  1.40 ± 1.50  2.30 ± 1.60  2.10 ± 1.60 | | | 2.80 ± 1.00  2.20 ± 1.00  2.80 ± 1.00  2.10 ± 1.00  2.80 ± 1.00  3.30 ± 1.00 | | | Swan et al. 2020 (Top Model) |
| COBU_TDF_CAM__RBC | | | RBC | | TDF_CAM_  (20-nest mean) | | | | 0.89 ± 0.67 | | | 1.90 ± 1.03 | | | Present Study |
| PEFA_CFE_FEATHER | | | Feather | | Controlled Feeding Experiment (Adult Peregrines) | | | | 2.10 ± 0.10 | | | 2.70 ± 0.50 | | | Hobson & Clark 1992 (Top Model in Swan et al. 2020) |
| COBU_TDF_CAM__FEATHER | | | Feather | | TDF_CAM_  (20-nest mean) | | | | 1.93 ± 0.79 | | | 3.03 ± 1.35 | | | Present Study |
| CACO_CFE_PLASMA | | | Plasma | | Controlled Feeding Experiment (Juvenile California Condors) | | | | 0.90 ± 0.20 | | | 3.30 ± 0.70 | | | Kurle et al. 2013 |
| COBU_TDF_CAM__PLASMA | | | Plasma | | TDF_CAM_  (20-nest mean) | | | | 0.19 ± 0.67 | | | 2.60 ± 1.11 | | | Present Study |

Table S4: Summary of nest camera diet data in the gyrfalcon (Johnson et al. 2020), peregrine (Robinson et al. 2018) and common buzzard (Swan et al. 2020) datasets. Data are displayed as mean (± 1SD).

| Study | # Cameras (# in high-quality subset) | Camera Type | Coverage Interval (days) | Nestling Age over Coverage Interval (days) | Mean Prey Biomass (daily rate per nestling in grams) | % Unk. Prey | Error Estimation Method |
| --- | --- | --- | --- | --- | --- | --- | --- |
| Gyrfalcon | 22 (2) | Photo  (Reconyx HyperFire) | 25.7  (± 3.1) | 0.4 (± 0.7) – 26.1(± 3.1) | 76.2 (± 47.3) | 0.7% | Bayesian bootstrap |
| Peregrine | 17 (3) | Photo  (Reconyx HyperFire) | 8.4  (± 3.3) | 2.8 (± 3.8) – 11.2 (± 3.5) | 87.6 (± 58.4) | 20.1% | Robinson et al. (2015) |
| Common Buzzard | 20 (NA) | Video  (CMOS 380 TVL) | 18.1  (± 7.1) | 22.3 (± 8.6) – 40.4 (± 4.5) | 53.4 (± 19.9) | 10.6% | Bayesian bootstrap |

Table S5: Methodological approach for attaining gyrfalcon covariates used as fixed effects in linear mixed models.

| Variable(s) | Methodological Approach |
| --- | --- |
| Hatch Date & Nestling Age | Motion-activated nest cameras were installed on gyrfalcon nest sites during mid-incubation (following Robinson et al. 2019), and continuously monitored the interval from their installation date to when nestlings were sampled for stable isotopes at ~25 days of age. Thus, hatch dates were recorded and exact nestling age could be determined. We defined a nest’s hatch date as the first date an egg hatched in the nest. Asynchronous hatch events were uncommon, and nestlings were typically the same age as their siblings +/- 2 days. |
| Nestling Sex | All gyrfalcon red blood cell samples used in the analysis were sexed genetically using p2/p8 primers at a commercial lab (Animal Genetics, Inc. Tallahassee, FL). |
| Body Condition | We generated an index of nestling body condition using the residuals from a regression of nestling body mass on an integrated measure of their structural size. When gyrfalcon nestlings were ~25 days of age, we visited nests to sample them for stable isotopes and also took several morphological measurements: p7 (feather) length, central retrix (feather) length, and tarsus length. We used a principal components analysis (PCA) to generate a measure of structural size for each sex individually, because gyrfalcons are sexually dimorphic (Booms et al. 2020). For females PC1 explained 93.8% of the variance, and for males PC1 explained 93.4% of the variance. For females, nestling mass was positively correlated with PC1 (OLS regression; β=102.81, p < 0.01, R-squared= 0.42). For males, nestling mass was also positively correlated with PC1 (OLS regression; β=74.30, p < 0.01, R-squared= 0.43). The resulting residuals of each regression were used as body condition estimates. |

Table S6: Ranking of gyrfalcon BSIMM agreement with nest camera dietary proportions, applying two different TDF_CAM_s: one incorporating all individuals in the dataset (GYRF_TDF_CAM__MEAN); and one incorporating individuals from two high-quality nests (GYRF_TDF_CAM__HQ; Table S1). Bhattacharyya’s coefficient (BC) is displayed for each prey category: PTAR (ptarmigan), AGSQ (Arctic ground squirrel), I. Bird (insectivorous bird), A.Rod (arvicoline rodent) and jaeger. The total BC values are the mean ± SD across categories.

| **Year** | **TDF** | **BC (Mean ± SD)** | **PTAR** | **AGSQ** | **I. Bird** | **A. Rod** | **Jaeger** |
| --- | --- | --- | --- | --- | --- | --- | --- |
| All | GYRF_TDF_CAM__MEAN | 0.757 ± 0.15 | 0.943 | 0.676 | 0.565 | 0.750 | 0.853 |
| All | GYRF_TDF_CAM_ _HQ | 0.738 ± 0.20 | 0.917 | 0.648 | 0.499 | 0.689 | 0.935 |
| 2017 | GYRF_TDF_CAM__MEAN | 0.552 ± 0.08 | 0.599 | 0.662 | 0.537 | 0.460 | 0.502 |
| 2017 | GYRF_TDF_CAM_ _HQ | 0.394 ± 0.10 | 0.448 | 0.538 | 0.300 | 0.304 | 0.381 |
| 2018 | GYRF_TDF_CAM__MEAN | 0.600 ± 0.09 | 0.660 | 0.646 | 0.442 | 0.579 | 0.672 |
| 2018 | GYRF_TDF_CAM_ _HQ | 0.547 ± 0.16 | 0.621 | 0.594 | 0.351 | 0.414 | 0.754 |
| 2019 | GYRF_TDF_CAM__MEAN | 0.800 ± 0.17 | 0.914 | 0.784 | 0.552 | 0.757 | 0.993 |
| 2019 | GYRF_TDF_CAM_ _HQ | 0.670 ± 0.26 | 0.890 | 0.608 | 0.356 | 0.522 | 0.974 |

Table S7: AIC output for linear mixed models testing the effects influencing gyrfalcon nestling TDF_CAM_s for carbon (∆^13^C) and nitrogen (∆^15^N). In all models, *territory* and *year* were included as random effects to account for non-independence. The most parsimonious model in each category is bolded. n=20 nest-years 2016-2019.

| **Response Variable** | **Explanatory Variables** | **K** | **AICc** | **∆AIC** | **AIC Wt.** |
| --- | --- | --- | --- | --- | --- |
| ∆^15^N | **% Ptarmigan + Body Condition + Age + Hatch Date** | **8** | **3.29** | **0.00** | **0.97** |
|  | % Ptarmigan + Body Condition + Age | 7 | 10.79 | 7.50 | 0.02 |
|  | % Ptarmigan + Body Condition + Age + Sex | 8 | 13.77 | 10.48 | 0.00 |
|  | % Ptarmigan + Body Condition | 6 | 15.59 | 12.30 | 0.00 |
|  | % Ptarmigan + Body Condition + Sex | 7 | 18.42 | 15.13 | 0.00 |
|  | % Ptarmigan | 5 | 48.52 | 45.23 | 0.00 |
|  | % Arvicoline Rodent | 5 | 57.48 | 54.20 | 0.00 |
|  | Body Condition | 5 | 61.06 | 57.77 | 0.00 |
|  | % Arctic Ground Squirrel | 5 | 70.27 | 66.99 | 0.00 |
|  | Age | 5 | 74.57 | 71.28 | 0.00 |
|  | % Jaeger | 5 | 87.66 | 84.38 | 0.00 |
|  | Hatch Date | 5 | 87.98 | 84.70 | 0.00 |
|  | Sex | 6 | 88.02 | 84.73 | 0.00 |
|  | Intercept-Only Model | 4 | 89.53 | 86.24 | 0.00 |
|  | % Shorebird | 5 | 91.89 | 88.60 | 0.00 |
| ∆^13^C | **% Arvicoline Rodent + Age + Sex** | **8** | **-22.64** | **0.00** | **0.79** |
|  | % Arvicoline Rodent + Age + Sex + Hatch Date | 9 | -19.88 | 2.76 | 0.20 |
|  | % Arvicoline Rodent + Age | 6 | -10.67 | 11.97 | 0.00 |
|  | % Arvicoline Rodent | 5 | -9.42 | 13.22 | 0.00 |
|  | Age | 5 | -9.02 | 13.62 | 0.00 |
|  | % Ptarmigan | 5 | -8.54 | 14.10 | 0.00 |
|  | % Arvicoline Rodent + Age + Body Condition | 7 | -8.47 | 14.17 | 0.00 |
|  | % Arvicoline Rodent + % Ptarmigan | 6 | -7.52 | 15.12 | 0.00 |
|  | % Arctic Ground Squirrel | 5 | -6.67 | 15.97 | 0.00 |
|  | Sex | 6 | 1.44 | 24.08 | 0.00 |
|  | % Shorebird | 5 | 8.38 | 31.02 | 0.00 |
|  | Body Condition | 5 | 11.04 | 33.68 | 0.00 |
|  | % Jaeger | 5 | 11.69 | 34.33 | 0.00 |
|  | Hatch Date | 5 | 13.29 | 35.93 | 0.00 |
|  | Intercept-Only Model | 4 | 14.34 | 36.98 | 0.00 |

Table S8: Ranking of peregrine BSIMM agreement with nest camera dietary proportions, comparing the incorporation of different TDFs and prior sets in the model. “I” is an informative prior (Table S2), and “U” is an uninformative prior. We tested four different TDFs: PEFA_CFE_BLOOD, PEFA_TDF_CAM__HQ, PEFA_TDF_CAM__MEAN, and COBU_TDF_CAM__PLASMA (Table S2). Bhattacharyya’s coefficient (BC) is displayed for each prey category: Duck (waterfowl), I. Bird (Insectivorous Bird), A.Rod (arvicoline rodent) and Seabird. The total BC values are the mean ± SD across categories.

| **Prior** | **TDF** | **Mean_BC** | **SD_BC** | **Duck** | **I. Bird** | **A. Rod** | **Seabird** |
| --- | --- | --- | --- | --- | --- | --- | --- |
| I | PEFA_TDF_CAM__MEAN | 0.833 | 0.068 | 0.832 | 0.765 | 0.809 | 0.927 |
| I | PEFA_TDF_CAM__HQ | 0.827 | 0.024 | 0.848 | 0.819 | 0.796 | 0.845 |
| I | COBU_TDF_CAM__PLASMA | 0.711 | 0.180 | 0.816 | 0.662 | 0.480 | 0.885 |
| U | PEFA_TDF_CAM__HQ | 0.645 | 0.151 | 0.852 | 0.611 | 0.491 | 0.627 |
| U | PEFA_TDF_CAM__MEAN | 0.630 | 0.158 | 0.836 | 0.518 | 0.493 | 0.671 |
| U | COBU_TDF_CAM__PLASMA | 0.568 | 0.259 | 0.894 | 0.445 | 0.293 | 0.639 |
| I | PEFA_CFE_BLOOD | 0.525 | 0.157 | 0.605 | 0.500 | 0.317 | 0.677 |
| U | PEFA_CFE_BLOOD | 0.402 | 0.372 | 0.859 | 0.123 | 0.076 | 0.549 |

Table S9: Ranking of common buzzard BSIMM agreement with nest camera dietary proportions, comparing the incorporation of different TDFs and prior sets in the model. We tested eight different TDFs across three tissue types: COBU_SIDER_BLOOD, COBU_TDF_CAM__RBC, GYRF_TDF_CAM__MEAN, PEFA_CFE_FEATHER, COBU_TDF_CAM__FEATHER, CACO_CFE_PLASMA, COBU_TDF_CAM__PLASMA, and PEFA_TDF_CAM__PLASMA (Table S3). Bhattacharyya’s coefficient (BC) is displayed for each prey category: A (amphibian), C (corvid), G (gamebird), I (insectivorous rodent); M (mouse) and R (rabbit). The total BC values are the mean ± SD across categories. The top model is bolded for each tissue type.

| **TDF** | **Mean**  **_BC** | **SD_BC** | **A** | **C** | **G** | **I** | **M** | **R** |
| --- | --- | --- | --- | --- | --- | --- | --- | --- |
| **COBU_TDF_CAM__RBC** | **0.693** | **0.106** | **0.549** | **0.762** | **0.620** | **0.743** | **0.647** | **0.839** |
| GYRF_TDF_CAM__MEAN | 0.634 | 0.136 | 0.572 | 0.718 | 0.497 | 0.643 | 0.520 | 0.857 |
| COBU_SIDER_BLOOD | 0.483 | 0.183 | 0.646 | 0.598 | 0.354 | 0.530 | 0.173 | 0.599 |
| **COBU_TDF_CAM__FEATHER** | **0.693** | **0.101** | **0.551** | **0.76** | **0.646** | **0.730** | **0.635** | **0.834** |
| PEFA_CFE_FEATHER | 0.512 | 0.108 | 0.593 | 0.776 | 0.506 | 0.743 | 0.580 | 0.721 |
| **COBU_TDF_CAM__PLASMA** | **0.692** | **0.102** | **0.555** | **0.759** | **0.628** | **0.716** | **0.651** | **0.842** |
| CACO_CFE_PLASMA | 0.555 | 0.137 | 0.643 | 0.611 | 0.323 | 0.553 | 0.487 | 0.711 |
| PEFA_TDF_CAM__PLASMA | 0.457 | 0.102 | 0.593 | 0.549 | 0.505 | 0.366 | 0.381 | 0.365 |

Figure S1. Mixing model output from applying the exact methodology used by Swan et al., 2020 (i.e., implementing SIAR rather than MixSIAR). Colored bars represent the proportional difference between common buzzard Bayesian stable isotope mixing model (BSIMM) diet estimates and nest camera diet estimates by prey category, comparing models constructed with two unique TDFs for red blood cell data: the top-performing TDF from the Swan et al., 2020 study (SIDER TDF) and the TDF_CAM_ this study calculated for common buzzard RBC (TDF_CAM_). Error bars are ± 1 SD. BC is the corresponding Bhattacharyya’s coefficient for each model.
